# Proximal Hypospadias Repair Outcomes in Patients with a Specific Disorder of Sexual Development Diagnosis

**DOI:** 10.1155/2012/708301

**Published:** 2012-06-20

**Authors:** Blake W. Palmer, William Reiner, Brad P. Kropp

**Affiliations:** Division of Pediatric Urology, The University of Oklahoma Health Sciences Center, 920 S. L. Young Boulevard, W.P. 3150, Oklahoma City, OK 73104, USA

## Abstract

Boys with undermasculinized external genital and/or 46,XY disorders of sex development (DSD) often receive masculinizing genitoplasty. Such procedures are done to correct ventral curvature of the phallus, reposition a proximally located urethral meatus, and cosmetically correct the appearance of labioscrotal folds. No studies to date have assessed if patients with a specific DSD diagnosis have worse outcomes for severe proximal hypospadias procedures or whether or not these patients require more extensive surgical maneuvers than severe proximal hypospadias patients without a specific DSD diagnosis. We retrospectively reviewed consecutive proximal hypospadias repairs performed at our institution from 1998 to 2010 and compared the anatomy, surgical technique required for repair, and outcomes in patients with and without a definitive DSD diagnosis. Boys with a specific DSD diagnosis do have significantly more atypical anatomy when undergoing proximal hypospadias masculinizing genitoplasties. They are more likely to require associated gonad procedures but do not have an increased risk of complications or number of surgeries when compared to other proximal hypospadias patients without a specific DSD diagnosis. The risk of complications is consistent with reports in the literature, and the mean number of procedures in this contemporary study is fewer than in historic reports.

## 1. Introduction

Boys with undermasculinized external genital development and/or a 46,XY disorder of sex development (DSD), including proximal hypospadias, often receive masculinizing genitoplasty. This procedure is done to correct ventral curvature of the phallus, reposition a proximally located urethral meatus, and cosmetically correct the appearance of external genitalia and labioscrotal folds. Need for genitoplasty can be associated with the need for such gonad operations as a biopsy when it will assist in making an accurate DSD diagnosis, gonadectomy in those cases at risk for gonadoblastomas and/or orchiopexy in those patients with associated cryptorchidism [1]. 

Although many recent reports have highlighted the dilemmas in accurately defining severe hypospadias, this anatomy would commonly be accepted as representing the most severe spectrum of the hypospadias complex [2, 3]. Despite a recent 20-year review of the severe hypospadias literature, no studies to date have assessed if patients with a definitive DSD diagnosis have worse outcomes for masculinizing genitoplasty procedures or whether or not these patients require more extensive surgical maneuvers than patients with severe proximal hypospadias not associated with a specific DSD diagnosis [4]. 

## 2. Materials and Methods 

We retrospectively reviewed consecutive proximal hypospadias repairs performed at our institution from 1998 to 2010. Patients were included if their initial meatal location was on the proximal portion of the shaft of the phallus or in a penoscrotal or perineal location. Patients were excluded if they had a distal or midshaft location of their hypospadias. Epidemiologic data was collected, including age at first surgery. Karyotype and specific DSD diagnosis were noted on all patients for whom this information was available. Anatomical data consisted of the meatal location at the time of surgery before intervention and gonad location at 9 months of age. Surgical data were collected regarding whether the procedure was planned to be done in 2 stages or not; if a urethral cutback was necessary because of deficient or dysplastic ventral skin and spongiosum; whether or not and how many Nesbit plication sutures were necessary to correct ventral angulation; whether or not the urethral plate was divided to correct ventral curvature; if corporal body grafting was done to correct ventral curvature; type of urethroplasty or scrotoplasty necessary; number and type of gonad procedures performed; number and type of postoperative complications; number and type of hypospadias procedures performed. Clinical data were assessed for length of follow-up and long-term complications.

The decision to stage the procedure and technique for orthoplasty and urethroplasty was at the discretion of the surgeon depending upon the child's anatomy as it presented itself during the initial surgical procedure. Decisions to stage a hypospadias procedure were related to the quality of the urethral plate and extent of curvature after skin degloving and ventral dissection of the corpora cavernosa that required division of the urethral plate to complete the orthoplasty portion of the procedure. In cases when the urethral plate was divided or required substitution, the procedure was staged.

Chi-square and unpaired Student's *t*-tests were used to compare differences between groups with and without a specific DSD diagnosis. Significance was determined if *P* < 0.05. 

## 3. Results

In this cohort, 102 patients were identified that met our study criteria with a urethral meatus in the proximal shaft, penoscrotal, or perineal location. A specific DSD diagnosis was identified in 17 patients (group 1), with mixed gonadal dysgenesis (MGD) in 35.3%, partial androgen insensitivity syndrome (PAIS) in 29.4%, other chromosomal abnormalities (3q12 addition and partial deletion of chromosome 1) in 11.8%, and Leydig cell aplasia, Klinefelter's variant, Opitz-Frias Syndrome, and VATER variant in 5.9% each (Table 1). The rest of the cohort without a specific DSD diagnosis was the comparison (group 2).

Table 2 compares the groups with a specific DSD diagnosis (group 1) and those without a specific DSD diagnosis (group 2). There were no significant differences between groups regarding age at initial surgery or percentage of either population with a proximal shaft or penoscrotal meatal location. Group 1 did have a significantly higher incidence of a perineal meatal location (70.6 versus 36.5%) and need for staging of the initial hypospadias procedure (58.8 versus 24.7%).

From an orthoplasty standpoint, group 1 was significantly more likely to require a division of the urethral plate to ultimately achieve a straight phallus (58.8 versus 23.5%). There was no difference seen in the incidence of Nesbit dorsal plications, number of these sutures required to correct the ventral curvature, or in the incidence of corporal body grafting for ventral lengthening when comparing the two groups. 

Group 2 was significantly more likely to have a tabularized incised plate (TIP) urethroplasty (35.3 versus 73.8%), whereas those in group 1 were more likely to have a scrotal skin tube inlay or onlay procedure (35.3 versus 9.5%). There was not a difference in the incidence of island inlay/onlay, flap inlay, or combination distal TIP/island inlay procedures. 

There was no difference seen in the incidence of simple or complex scrotoplasties to achieve a masculinized external appearance of the labial scrotal folds and correct scrotal transposition when comparing groups.

 Group 1 had a significantly higher incidence of associated undescended testicles requiring orchiopexy (47.1 versus 14.1%), incidence of gonad biopsy (23.5 versus 0.0%), and incidence of gonadectomy (35.3 versus 0.0%) when compared to the group 2.

There were no significant differences between groups in mean total number of procedures required, mean total number of procedures minus planned staged procedures, number of patients with a postoperative complication, or length of clinical follow-up between the groups. 

## 4. Discussion

Although most boys with a diagnosis of 46,XY DSD with an undermasculinized phallus and scrotum require proximal hypospadias repairs to masculinize their external genitalia, to our knowledge, this is the first paper to show that having a specific DSD diagnosis does not result in worse surgical outcomes. The two groups are similar in age at surgery in this paper. The trend toward an older age at initial surgery in the group with a specific diagnosis (group 1) could be related to delay in definitive care to ensure a full DSD evaluation is completed and the parents have decided upon a sex of rearing. One patient in group 1 was an outlier for age at initial surgery for this reason and was not operated on until after puberty. 

It is unclear which patients with a proximal hypospadias presentation should have a DSD evaluation, how extensive this evaluation should be, and whether or not all hypospadias patients should be considered a DSD diagnosis [2]. Although the 2006 consensus proposes considering all patients with a congenital abnormality as being classified as DSD, at our institution, any patient with a proximal urethral meatus location and/or severe ventral curvature with an associated undescended testicle would have a DSD evaluation by the pediatric endocrinology team, beginning with a karyotype and a testosterone level (if age 15–90 days within the minipuberty period or after HCG stimulation if older) [1, 2, 5]. If all results are normal, the patient would be considered to have an idiopathic proximal hypospadias without a specific DSD diagnosis. If there were abnormalities in the chromosome or hormonal profile, a specific diagnosis would be investigated further by our multidisciplinary DSD team. This would be done to give the providers, family, and, ultimately, the patient the best information available as to the long-term expectations for (1) the need for hormone supplementation at puberty, (2) surgical options to make the external genitalia consistent with the sex of rearing, (3) potential for fertility, (4) potential cancer risk, and (5) stability of sex of rearing with aging. Historically only about 50% or less of 46,XY DSD patients receive a specific diagnosis but with this approach we have achieved a current rate of 69% at our institution [1]. Identifying and classifying these patients accurately will help us to better understand the associated pathophysiology and effects on surgical outcomes. The results of this paper will assist us in more accurately preparing our surgery team for these complex procedures and provide more accurate expectations for families and patients.

Limitations of this paper are the small sample size from a single institution with a somewhat heterogeneous population. Other confounding factors include significant differences in the type of urethroplasty performed to correct these severe defects, although there was not a difference in the number of patients who had a postoperative complication. The incidence of complications in both groups was consistent with that reported in the literature, as evidenced by a comprehensive review of 2,203 proximal hypospadias patients over the past 20 years that revealed a complication rate of 14.9–45.7% [4]. Our complications rate is superior to long-term outcomes, showing a 51% fistula rate in masculinizing genitoplasty procedures for patients with DSD as reported after 2-stage Dennis Browne techniques [6]. This is despite the fact that group 1 received more complicated surgical procedures to correct the urethral position when compared to the patients in group 2. 

The mean number of procedures for patients in group 1 was not significantly different from that of group 2, nor was the mean number of procedure after controlling for the increased incidence of planned 2-stage procedures. This mean of 2.06 procedures is fewer than the number of masculinizing genitoplasty procedures reported in a historic cohort of males with 46,XY DSD who received genitoplasty between 1950 and 1990 [7]. 

Despite the limitations of this study, we believe that a consistent systematic DSD evaluation should be done for all proximal hypospadias boys. This will help surgeons identify those patients who are more likely to require staged reconstructions and more complicated procedures during their masculinizing genitoplasty. This knowledge also is potentially helpful to families in counseling long-term and further genetic evaluation when appropriate. This also contributes to the literature highlighting the importance of studying the effects to optimize the most appropriate workup for these patients and the surgical outcomes that can be seen and stratified by specific diagnosis once the workup is best able to distinguish one patient from another. 

## 5. Conclusions

Boys with a specific DSD diagnosis do have significantly more atypical genital anatomy prior to receiving surgeries for their proximal hypospadias than do 46,XY boys with proximal hypospadias without a specific DSD diagnosis, and they also are more likely to require associated gonad procedures. However, these boys do not have an increased risk of surgical complications or number of surgeries received. The risk of complications associated with genitoplasty in our cohort is consistent with contemporary reports in the literature and superior to historical reports indicating improvements in surgical outcomes over time.

## Figures and Tables

**Table 1:** Distribution of specific DSD diagnoses.

Diagnosis	Number	Percentage
45XO/46XY mixed gonadal dysgenesis	6	35.3
46XY PAIS	5	29.4
46XY Leydig cell aplasia	1	5.9
46XXY Klinefelter's variant	1	5.9
Other Chromosomal Abnormalities	2	11.8
Opitz-Frias syndrome	1	5.9
VATER	1	5.9

	*n* = 17	

**Table 2:** Anatomy, procedure, and outcomes comparison.

	Group 1: proximal hypospadias with a specific DSD diagnosis (*n* = 17)	Group 2: 46,XY proximal hypospadias without a specific DSD diagnosis (*n* = 85)	*P* value
Mean age at initial hypospadias repair (years)	2.37	1.53	*P* = 0.437

Meatal location			
Proximal shaft	2 (11.8)	16 (18.8)	*P* = 0.730
Penoscrotal	3 (17.6)	38 (44.7)	*P* = 0.056
Perineal	12 (70.6)	31 (36.5)	*P* < 0.05
Planned stage procedure	10 (58.8)	21 (24.7)	*P* < 0.05

Orthoplasty			
Nesbit plication	16 (94.1)	72 (84.7)	*P* = 0.455
Mean number of Nesbit plications sutures	1.25	1.32	*P* = 0.686
Division of urethral plate	10 (58.8)	20 (23.5)	*P* < 0.05
Corporal body grafting	2 (11.8)	11 (12.9)	*P* = 1.000

Urethroplasty			
TIP	6 (35.3)	62 (73.8)	*P* < 0.05
Scrotal skin tube inlay/onlay	6 (35.3)	8 (9.5)	*P* < 0.05
Island inlay/onlay	2 (11.8)	3 (3.6)	*P* = 0.196
Flap inlay	2 (11.8)	3 (3.6)	*P* = 0.196
Combination distal TIP/island inlay	1 (5.9)	8 (9.5)	*P* = 0.705

Scrotoplasty			
Simple	4 (25)	31 (37.8)	*P* = 0.402
Complex	12 (75)	51 (62.2)	*P* = 0.402

Gonads			
UDT	8 (47.1)	12 (14.1)	*P* < 0.05
Gonad biopsy	4 (23.5)	0 (0.0)	*P* < 0.05
Gonadectomy	6 (35.3)	0 (0.0)	*P* < 0.05

Outcomes			
Mean total number of procedures	2.06	1.61	*P* = 0.119
Mean total number of procedures—planned procedures	0.47	0.36	*P* = 0.669
Patients with a complication	5 (29.4)	19 (22.4)	*P* = 0.756
Clinical follow-up (years)	2.07	1.89	*P* = 0.732
